# Successful incorporation of single reviewer assessments during systematic review screening: development and validation of sensitivity and work-saved of an algorithm that considers exclusion criteria and count

**DOI:** 10.1186/s13643-021-01632-6

**Published:** 2021-04-05

**Authors:** Nassr Nama, Mirna Hennawy, Nick Barrowman, Katie O’Hearn, Margaret Sampson, James Dayre McNally

**Affiliations:** 1 Faculty of Medicine, University of British Columbia, Vancouver, BC Canada; 2 Pediatrics, BC Children’s Hospital, Vancouver, BC Canada; 3 Faculty of Medicine, University of Ottawa, Ottawa, ON Canada; 4 Pediatrics, Children’s Hospital of Eastern Ontario, 401 Smyth Road, Ottawa, Ontario K1H 8L1 Canada; 5 Clinical Research Unit, CHEO Research Institute, Ottawa, ON Canada

**Keywords:** Systematic review, Exclusion criteria, Rapid reviews, Citation screening, Single-reviewer

## Abstract

**Background:**

Accepted systematic review (SR) methodology requires citation screening by two reviewers to maximise retrieval of eligible studies. We hypothesized that records could be excluded by a single reviewer without loss of sensitivity in two conditions; the record was ineligible for multiple reasons, or the record was ineligible for one or more specific reasons that could be reliably assessed.

**Methods:**

Twenty-four SRs performed at CHEO, a pediatric health care and research centre in Ottawa, Canada, were divided into derivation and validation sets. Exclusion criteria during abstract screening were sorted into 11 specific categories, with loss in sensitivity determined by individual category and by number of exclusion criteria endorsed. Five single reviewer algorithms that combined individual categories and multiple exclusion criteria were then tested on the derivation and validation sets, with success defined a priori as less than 5% loss of sensitivity.

**Results:**

The 24 SRs included 930 eligible and 27390 ineligible citations. The reviews were mostly focused on pediatrics (70.8%, *N*=17/24), but covered various specialties. Using a single reviewer to exclude any citation led to an average loss of sensitivity of 8.6% (95%CI, 6.0–12.1%). Excluding citations with ≥2 exclusion criteria led to 1.2% average loss of sensitivity (95%CI, 0.5–3.1%). Five specific exclusion criteria performed with perfect sensitivity: conference abstract, ineligible age group, case report/series, not human research, and review article. In the derivation set, the five algorithms achieved a loss of sensitivity ranging from 0.0 to 1.9% and work-saved ranging from 14.8 to 39.1%. In the validation set, the loss of sensitivity for all 5 algorithms remained below 2.6%, with work-saved between 10.5% and 48.2%.

**Conclusions:**

Findings suggest that targeted application of single-reviewer screening, considering both type and number of exclusion criteria, could retain sensitivity and significantly decrease workload. Further research is required to investigate the potential for combining this approach with crowdsourcing or machine learning methodologies.

**Supplementary Information:**

The online version contains supplementary material available at 10.1186/s13643-021-01632-6.

## Background

Conducting systematic reviews (SRs) is labour-intensive and often requiring up to several years for completion [1, 2]. Given this lengthy period for review completion, close to 25% of SRs are out of date within 2 years due to emergence of new evidence [3]. The need for faster turnaround times has been emphasized in the context of the current COVID-19 pandemic [4, 5]. Citation screening is one of the most time-intensive steps in the review process [6]. The accepted gold-standard approach is having at least two reviewers independently screen abstracts and full-text citations and resolving any discrepancies through consensus or a third party [7].

There is a growing body of evidence to support the use of innovative methodologies in this step, such as crowdsourcing, machine learning or data mining [8–11]. New adjuncts to reduce the time spent on this step will need to consider the effects of efficiency on accuracy. Another approach to expedite the citation screening process and decrease the workload on the investigative team is through screening by a single reviewer. However, this approach is generally considered too high risk when applied to all citations [12–15], leading to a loss in recall of citations up to 13%, with a substantial change in the findings for 43% of the reviews [16].

However, it is unlikely that all eligible citations share an equal risk of being misclassified by reviewers, due to differences in quality and content of title and abstract preparation and subjectivity in the application of different inclusion and exclusion screening criteria.

A few research studies have described the types and prevalence of exclusion criteria [17, 18], yet none have evaluated if some are more likely to cause disagreement between the two reviewers [12]. Improved understanding of errors associated with specific exclusion criteria has the potential to decrease the workload, by requiring a second reviewer only in instances where the exclusion criteria identified have unacceptable disagreement rates. First, we hypothesized that when a single reviewer identifies multiple distinct exclusion reasons in a citation, exclusion without a second review will lead to minimal loss in sensitivity. Second, we hypothesized that there would be specific exclusion criteria that are sufficiently objective to allow single-review assessment and thus forgo confirmation by a second reviewer.

The objectives of this study are to (i) compare the loss in sensitivity during single review screening between citations identified as having one versus multiple exclusion criteria, (ii) determine the loss in sensitivity with single-reviewer screening when considering application of specific categories of exclusion criteria, and (iii) develop and validate algorithms that utilize various combinations of individual and multiple exclusion criteria, with the goal of minimizing loss of sensitivity and maximizing work saved.

## Methods

### Study design

This study was focused on the following forms of systemic synthesis of evidence (systematic reviews, scoping reviews, meta-analysis, living reviews). For simplicity, the term systematic review is used in this paper. Citation screening proceeded as per the gold-standard approach of two independent assessments per citation. In this retrospective study, we determined the sensitivity and work-saved that would have been obtained with the application of a single-reviewer assessment under a variety of circumstances. Results are reported according to the Standards for Reporting of Diagnostic Accuracy Studies guidelines (Additional table 1) [19].

### Systematic review selection and details

Systematic reviews completed at CHEO since 2016 and screened using the insightScope platform (previously called CrowdScreen) were eligible for this study [9]. CHEO is the sole pediatric health care and research centre in Ottawa and is affiliated with the University of Ottawa. Only SRs that were screened in duplicate by independent experts or trained reviewers were eligible. For each SR, the following was retrieved from the platform: (1) the full list of citations screened by the investigative team, (2) the inclusion and exclusion criteria, (3) the final list of citations determined to be eligible by the expert reviewers (true positives) and (4) the reason(s) either reviewer excluded a citation. SRs completed between January 2016 and December 2017 using the CrowdScreen platform were used for the derivation set, and those completed between January 2018 and December 2020 on the insightScope platform were used for the validation set. The analysis was focused on screening at the abstract level.

### Univariate analysis of exclusion criteria type and number

The exclusion criteria provided by the investigative teams were placed in one of the following categories: (1) conference abstract, (2) review article (i.e. the systematic review only selected primary research), (3) ineligible language, (4) not human research, (5) ineligible population, (6) ineligible age group, (7) ineligible exposure/intervention, (8) ineligible outcome, (9) ineligible setting, (10) case reports/series and (11) ineligible design (e.g. not a randomized controlled trial). Examples of each exclusion criterion are provided in supplements (Additional table 2).

For each exclusion criterion, we then determined the loss in sensitivity that would have occurred with application of this criterion (alone) in a single assessment setting. Loss of sensitivity was calculated as the percentage of the final eligible citations (true positives) where the first reviewer excluded (false negative), while selecting this criterion. The order of the two independent assessments within each citation was randomized. This randomization was repeated 1000 times to obtain a mean and standard deviation (SD) for each outcome of interest. Wilson’s 95% confidence interval (95%CI) was generated around the mean.

Reviewers were able to select one or multiple exclusion criteria. They were not instructed to select all applicable reasons for exclusion. A similar analysis was completed using the number of exclusion criteria selected in each citation. For each threshold between 1 and 4, we included citations that had that number of exclusion criteria or more. The number of eligible citations excluded by one reviewer with one reason of exclusion was compared to citations excluded while selecting two or more reasons, using a Pearson’s chi-square test.

### Algorithm development and validation

The exploratory analysis informed the development of 5 algorithms. These relied on excluding a citation by a single reviewer, when their assessment indicated multiple exclusion reasons and/or selected a criterion that had shown a loss of sensitivity of either 0% or <1% in univariate analysis (Table 1, Additional figure 1). Evaluation of the algorithms proceeded as follows. First, each algorithm was applied to the citations in the derivation set. As each citation had two assessments, these were randomly ordered. This randomization process was repeated 1000 times to obtain a mean and SD for each outcome. Wilson’s 95% confidence interval (95%CI) was generated around the mean. The algorithm was applied to the first assessment, when its reviewer has chosen to exclude the citation. If the number of and/or specific exclusion criteria selected satisfied an algorithm’s conditions, the citation was excluded. If not, the assessment of a second reviewer was taken into account to determine if the record would be retained or excluded. Similar to the gold-standard two-reviewers approach and in cases of disagreement, a third reviewer or the principal investigator decided on the outcome for that citation.

**Table 1:** Description of five algorithms developed based on the exploratory analysis

	≥ 2 exclusion criteria	Exclusion criterion with loss of sensitivity ≤ 1%	Exclusion criterion with no loss in sensitivity
**Algorithm 1**	X		
**Algorithm 2**		X	
**Algorithm 3**			X
**Algorithm 4**	X	X	
**Algorithm 5**	X		X

The abstracts retained using each algorithm were compared to the final list of true positives as provided by the principal investigators, for calculation of loss of sensitivity and work-saved to the investigative team. Work-saved to the second reviewer was defined as the percentage of all citations that were excluded by the algorithm using a single reviewer.

There is no clearly defined threshold for acceptable sensitivity in the literature; however, multiple studies in field of innovative methodology of SR screening have used a sensitivity of 95% as cutoff [20, 21]. It was decided a priori that algorithms achieving a loss of sensitivity below 5%, point estimate and upper 95%CI, were to be tested on the SR validation set.

### Evaluation of eligible studies missed by the algorithms

To investigate potential differences between citations at risk for being missed using a single-reviewer approach, citations missed by any algorithm (false negatives) were compared to eligible citations not missed by any of the algorithms (true positive). True positives were randomly selected from the same SR as the false negatives and matched 1:2. SRs, from both the derivation and validation sets were included for this analysis. The following measures were compared: (1) the citation’s year of publication, (2) the journal’s impact factor closest to the time of publication (based on the Journal Citation Reports), (3) the number of times the individual paper was cited as on May 23 2020 (based on Web of Science), (4) the study size based on number of patients or study participants, (5) whether an abstract was structured and (6) whether the citation was available on PubMed. For continuous outcomes, the two groups of citations (false negatives and true positives) were compared using a Wilcoxon test. Binary outcomes were compared using a Fisher’s exact test.

### Data analysis

Data analysis was done in R (version 4.0.0).

## Results

### Description of evaluated systematic reviews

In total, 24 reviews were evaluated in this study (Table 2, Additional table 3) including 21 systematic reviews (14 of which involved a meta-analysis) and 3 scoping reviews. Reviews screened a median of 555 abstracts (range: 232–9648) and 119.5 full-texts (range: 15–453), and identified a median of 26 eligible citations (range: 2–159). The reviews were mostly focused on pediatrics (70.8%, *N*=17/24), but covered a wide variety of specialties (Table 2).

**Table 2:** Description of included systematic reviews

	Derivation set	Validation set
Systematic reviews, *N* (%)	10 (42%)	14 (58%)
Type of review, *N* (%)		
Systematic review without meta-analysis	7 (70%)	7 (50%)
Systematic review with meta-analysis	2 (20%)	5 (36%)
Scoping review	1 (10%)	2 (14%)
Living review	0 (0%)	4 (29%)
Focus		
Therapeutic	3 (30%)	8 (57%)
Diagnosis	3 (30%)	2 (14%)
Prognosis	1 (10%)	1 (7%)
Other	3 (30%)	3 (21%)
Screening, median (range)		
Abstract screened	484 (257 - 2469)	745.5 (232–9648)
Full-text screened	110 (15 - 441)	150 (16–453)
Eligible citations	27 (9 - 68)	21 (2–159)
Reviewers	3 (2 - 6)	5 (2–13)
Kappa	0.47 (0.25 - 0.76)	0.56 (0.37–0.9)
Field of study, *N* (%)		
Pediatrics	8 (80%)	9 (64%)
Respirology	2 (20%)	1 (7%)
Development	2 (20%)	0 (0%)
Public Health	1 (10%)	5 (36%)
Pathology	1 (10%)	3 (21%)
Emergency	1 (10%)	1 (7%)
Medical Education	1 (10%)	1 (7%)
Surgery	1 (10%)	1 (7%)
Neonatology	1 (10%)	0 (0%)
Nursing	1 (10%)	0 (0%)
Oncology	1 (10%)	0 (0%)
Psychiatry	1 (10%)	0 (0%)
Radiology	1 (10%)	0 (0%)
Cardiology	0 (0%)	1 (7%)
Critical care	0 (0%)	1 (7%)

Type of review and field of study were not exclusive, as studies can cover multiple choices. Numbers add up to more than 100%

**Fig. 1:**
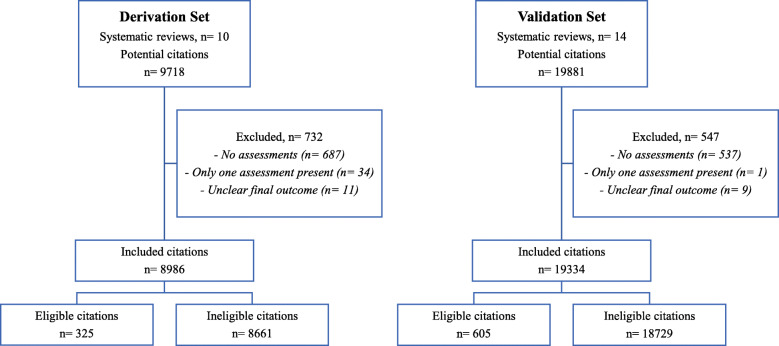
Flow diagram of included citations in the derivation and validation sets

For the derivation set, there were 9718 citations across the ten systematic reviews, with 8986 (92.5%) having been screened in duplicate and having final eligibility available (Fig. 1). Citations were screened by a total of 34 independent reviewers, with an inter-rater reliability (kappa) of 0.49. Of the 8986 citations, 325 (3.6%) were eligible and 8661 were ineligible.

For the validation set, the 14 systematic reviews contained 19881 citations, with 19334 (97.2%) having been screened in duplicate and included in the analysis (Fig. 1). Citations were screened by a total of 82 independent reviewers, with an overall inter-rater reliability (kappa) of 0.70. Of the 19334 citations, 605 (3.1%) were eligible and 18729 were found to be ineligible, by the gold-standard two-reviewers approach.

### Development set exclusion criteria evaluation and algorithm testing

In the derivation set, 56 (17.2%) of the 325 true positives (eligible) citations were excluded by one reviewer (false negative). The average loss of sensitivity for single-review assessment was calculated to be 8.6% (95%CI, 6.0–12.1%).

**Fig. 2:**
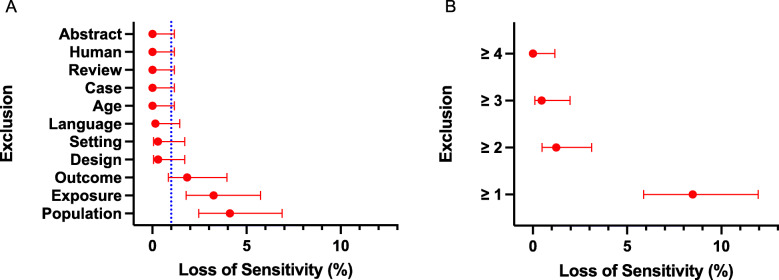
Loss of sensitivity when permitting single reviewer exclusion, based on specific criteria. Error bars reflect 95%CI. The blue dotted line reflects the 1% threshold used in the algorithm development stage. Analysis is based on the systematic reviews in the derivation set only

Eleven exclusion criteria were evaluated for their individual performance. Point estimates for loss of sensitivity ranged between 0 and 4.1%. The following five exclusion criteria resulted in 0 false negatives, that is, had a 0% loss of sensitivity with single-reviewer screening: conference abstract, ineligible age group, case report/series, not human research and review article (Fig. 2, Additional table 4). There were three additional exclusion criteria with average loss of sensitivity under 1%: ineligible language 0.2%, ineligible design 0.3% and ineligible setting 0.3%.

In the 8986 analyzed citations in the derivation set, 48.4% (*N*=4347) were excluded while providing one reason of exclusion (Additional table 5). Single reviewer single criteria exclusion of citations account for 7.2% (95%CI, 4.9–10.5%) of the 8.6% loss in sensitivity (Table 3). For the 3674 citations with ≥2 exclusion criteria selected, they accounted for 1.2% (95%CI, 0.5–3.1%) of the loss in sensitivity; the difference in sensitivity loss between those two groups was highly statistically significant (*P*<0.001). We observed no loss of sensitivity in cases where four or more exclusion criteria were met.

**Table 3:** Loss of sensitivity when permitting single reviewer exclusion, based on number of unique criteria

Exclusion ^**a**^	Count of SRs	Count of Papers ^**b**^	Loss of Sensitivity Mean [95%CI]
≥ 1	10	8021	8.4% [5.9–12.0%]
≥ 2	8	3674	1.2% [0.5–3.1%]
≥ 3	8	1449	0.5% [0.1–2.0%]
≥ 4	6	296	0.0% [0.0–1.2%]

^a^Analysis is based on the systematic reviews in the derivation set only

^b^Number of papers where the criterion was selected

### Algorithm derivation

**Fig. 3:**
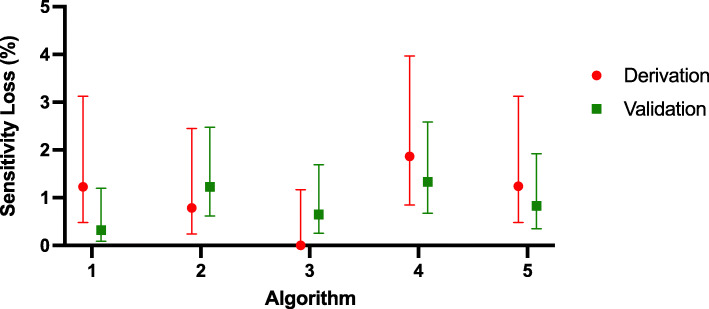
Loss of sensitivity of algorithms employing a single reviewer approach. Loss of sensitivity is the percentage of eligible citations incorrectly excluded by the algorithm at the abstract level among all eligible citations. Error bars reflect 95%CI. Analysis is based on the systematic reviews in the derivation set (red) and the validation set (green)

Five algorithms were developed as described in the methods and evaluated initially using the SR derivation set (Table 1, Additional figure 1). In the derivation set, the five algorithms resulted in loss of sensitivity ranging from 0.0% to 1.9% (Fig. 3, Additional table 6), with work saved ranging from 14.8 to 39.1% (Table 4). The upper end of the 95%CI of loss of sensitivity was below the a priori defined acceptable threshold of 5% for all five algorithms. The most conservative algorithm (3), with no loss of sensitivity (0.0%, 95%CI, 0.0–1.2%), demonstrated the lowest work-saved 14.8% (95%CI, 14.1–15.6%). Conversely, algorithm 4 with the highest work-saved 37.7% (95%CI, 36.7–38.7%) also had the highest loss in sensitivity 1.9% (95%CI, 0.8–4.0%).

**Table 4:** Work-saved by algorithms employing a single reviewer approach

Algorithm	Work-saved (%) ^**a**^ Mean [95%CI]	
	Derivation	Validation
1	25.2% [24.3–26.1%]	32.0% [31.4–32.7%]
2	22.2% [21.3–23.0%]	27.5% [26.8–28.1%]
3	14.8% [14.1–15.6%]	10.5% [10.1–10.9%]
4	37.7% [36.7–38.7%]	48.2% [47.5–48.9%]
5	33.6% [32.6–34.5%]	39.8% [39.1–40.5%]

^a^Work-saved is defined as the percentage of all citations that were excluded by the algorithm without requiring a second assessment by the investigative team

**Table 5:** Comparing eligible citations retained and missed by derived algorithms

Variable	True positives	False negatives	*p* value
*N*	55	28	
Year, median (IQR)	2012 (2005–2015)	2014 (2009–2016)	0.57
Population size, median (IQR)	178 (23–640)	303 (72–901)	0.14
Citations, median (IQR)	13 (5–26)	11 (6.5–25)	0.96
Journal impact, median (IQR)	2.79 (2.12–3.98)	3.03 (1.88–3.83)	0.93
Structured abstract, *N* (%)	32 (58.2%)	10 (35.7%)	0.07
Available on PubMed, *N* (%)	51 (92.7%)	27 (96.4%)	0.66

### Algorithm validation

The performance of these five algorithms was validated on the second set of systematic reviews (Fig. 3, Table 4). Compared to the derivation set, the average loss of sensitivity of each algorithm was similar (−0.9 to 0.6%). The 95% confidence interval of loss of sensitivity remained well below our a priori threshold for all five algorithms (maximum loss of 2.6% in algorithm 4). The average work-saved for the five algorithms varied between 10.5% and 48.2%. Algorithm 4 remained the one with the highest work-saved 48.2% (95%CI, 47.5–48.9%), and the highest loss of sensitivity 1.3% (95%CI, 0.7–2.6%).

### Evaluation of eligible studies missed by the algorithms

Overall, 28 eligible studies were missed by one or more of the algorithms (false negatives). Comparing those studies with citations accurately identified by all five algorithms did not identify a significant difference (Table 5) in year of publication (*p*=0.57), population size (*p*=0.14), number of papers citing this study (*p*=0.96), the journal impact factor (*p*=0.93), whether abstract was structured (*p*=0.07) or whether the citation was available on PubMed (*p*=0.66).

## Discussion

This study demonstrated that across a large and diverse collection of systematic reviews, adopting a non-targeted, single-reviewer screening strategy would have missed 9% of eligible studies. As hypothesized, evaluation of type and number of exclusion criteria demonstrated substantial variability in loss of sensitivity. By defining specific types and combinations of exclusion criteria, we developed five algorithms for single-reviewer assessments with minimal loss of sensitivity (< 2%). Performance of these algorithms was validated on a separate set of SRs; all five algorithms had sensitivity values above 98%, with reduction of approximately 10–50% of the work for the second assessor.

This study showed a high variability in the prevalence of individual exclusion criteria, and their associated sensitivity and specificity. While no other studies have evaluated loss in sensitivity by exclusion reason, others have reported on the prevalence of exclusion reason [17, 18]. For example, Edinger et al. showed that commonly selected reasons for exclusion were ineligible design (65%), ineligible intervention (14%) and ineligible outcome (11%) [18]. In our study, the most common exclusion criteria were somewhat different: ineligible population (59%), ineligible exposure (58%) and ineligible outcome (35%). Additionally, our study found that reviewers were more prone to incorrectly exclude a citation based on the diagnosis, exposure and outcome of interest. This suggests that investigative teams should particularly focus on defining these criteria clearly [16]. Interestingly, the specific criteria with worse prediction value differed in these algorithms compared to machine learning approaches. A recent study on machine learning revealed a propensity for incorrect prediction in identifying observational studies, reviews, studies with low risk of bias and older citations [22]. Additionally, we found a significant decrease in loss of sensitivity when two or more exclusion criteria were selected. While this aspect has not been assessed previously in the literature, a comparable example maybe found in natural language processing [23]. For example, when using the “bag of words” model, particular words are assigned different weights/coefficients to reflect the likelihood of abstract inclusion/exclusion. These coefficients are cumulative; hence, the more exclusion words present, the higher the likelihood of the citation being ineligible [23].

Identification and inclusion of all relevant studies on a topic is considered critical to completion of an unbiased systematic review. To achieve this goal, the field has adopted dual independent screening as the gold standard, with good supporting evidence demonstrating an increase in recall compared to single-reviewer assessment [12–15]*.* Consequently, and often appropriately, journals and readers should be wary of reviews that only use a single reviewer for screening and/or data extraction. Nonetheless, when faced with large citation set sizes and limited resources or time constraints some teams make the decision to incorporate single reviewer screening (e.g., rapid reviews) [24, 25]. Readers are then forced to decide the extent with which this methodological decision and approach undermines the findings. Similar to other studies on single-reviewer assessments, we calculated a 9% loss in sensitivity for the non-targeted single reviewer method [16, 26]. A recent SR lead by Waffenschmidt comparing single- and dual-reviewers approaches detected a median loss of sensitivity of 5% (range 0–58%), with a substantial change in the results of meta-analyses in almost half of the analyzed studies [16]. Another study by Stoll et al. reported that a completely dual-reviewer approach identified an additional 6.6–9.1% of eligible studies [26]. Our study is the first to evaluate single-reviewer assessments using a targeted approach, demonstrating substantially improved recall of eligible studies. Five algorithms showed small loss of sensitivity with an upper limit of the confidence interval below 3% in the validation phase. This presents an appealing adjunct to the rapid review methodology by significantly reducing the workload while limiting data loss. Other strategies employed for rapid reviews have relatively larger loss of sensitivity. For example, limiting the search to PubMed only led to the loss of all identified studies in 3.7% of Cochrane reviews, and a moderate to large change in the primary outcome in 10% [27]. Meanwhile, limiting the search to the last 20 years led to a loss of all studies in 10% of reviews, and a moderate to large change in the effect estimate in 21%.

While preserving sensitivity is critical, this approach is only valuable if there is a meaningful reduction in workload. The highest work-saving algorithm (the fourth) reduced the second reviewer’s workload by almost half, with only 1.3% loss of sensitivity. Despite the minimal variation in loss of sensitivity among the algorithms (0 to 2%), those with higher retention of eligible citations showed lower work-saved. As algorithm 4 achieved both the a priori threshold for loss of sensitivity and significant workload reductions, it may seem redundant to report the findings for the other algorithms. However, given the lack of consensus on an acceptable loss of sensitivity, some researchers may want to consider even more conservative approaches. For example, in fields with a small number of trials, the authors might prioritize sensitivity to maximize the number of identified eligible studies. Conversely, in areas requiring a broader search, reducing workload might be a priority. Considering the review on perinatal infections, which was largest in this analysis, the algorithm prioritizing work-saved reduced the second reviewer’s workload by 51.8% of the abstracts (*N*=4953/9648). With citation screening requiring on average 1 min and a cost of $1.50 [15], this saves approximately 83 h of screening and $7500 while still identifying 66/68 eligible studies. Compared to our findings, machine learning methodologies have shown higher potential to reduce work, provided that researchers are skilled in using that methodology [28]. However, loss of sensitivity was more pronounced with machine learning than our approach [11]. Gates et al. reported that automation of screening in 11 SRs resulted in a 0–38% loss of eligible studies and that 28–85% of the citations were excluded without input from the investigative team [29]. Meanwhile, Tsou et al. compared commonly used tools, EPPI-Reviewer and Abstrackr [30]. They reported that approximately 30–70% of citations required screening by the investigative team to train the model to achieve a sensitivity exceeding 95% on three large SRs. Alternatively, studies using crowdsourcing have demonstrated high sensitivity (86–100%), depending on the number of assessments per abstract [8–10, 31, 32]. Depending on the crowdsourcing approach, the investigative team’s workload was reduced by 45–60% [8]. The algorithms identified herein have shown comparable, if not higher, sensitivity. The two strategies can potentially be combined. Most crowdsourcing approaches require three or four independent crowd members to review an abstract [33]. Using these algorithms, this number may be further lowered depending on the type and number of exclusion criteria selected. However, our current results are based on expert reviewers and may not generalize to non-expert reviewers.

The strengths of this study stem from the evaluation of data from a large number of SRs covering a wide variety of research topics. This is the first study to assess human reviewers’ error rates during abstract screening based on specific exclusion criteria. Furthermore, the results of the algorithms showed consistent performance when validated on a separate set of SRs. Hence, these algorithms offer additional strategies in the toolbox to conduct SRs more efficiently. This approach will also decrease the number of full-text citations to be retrieved and screened by both reviewers [23]. Additionally, the reviewers in our study were not instructed to select all applicable criteria. Reviewers’ awareness of the algorithm might prompt them to indicate multiple exclusion reasons, when applicable, and further reduce the workload. Finally, the loss of sensitivity reported herein is likely overestimated. Missed citations might be identified at subsequent stages of the review process, such as while reviewing the reference lists from eligible studies and contacting their authors [34].

The limitations of this study include the inability to stratify results based on an individual reviewer’s expertise, as screening was performed by subject experts in these reviews. Additionally, this study focused on abstract screening, and the results are not generalizable to the screening of full-text citations. The study did not assess the benefits of moving abstracts retained by a single reviewer to the full-text stage, without obtaining a second assessment, a method known as liberal acceleration [35, 36]. While this might reduce the amount of work saved at the abstract level, it could increase the work required to retrieve and screen these additional citations at the full-text level. A limitation or barrier to adoption and wide-spread application of a targeted single review approach to screening may be the desire for validation in the local setting at either the institute or individual study level. To assist with this, we have provided a recommended list of steps (Additional table 7).

## Conclusions

In conclusion, given a confirmed sensitivity between 98 and 100% across a large number of systematic reviews, a targeted single-reviewer abstract-screening approach may be acceptable in circumstances where a citation is ineligible for multiple reasons or based on more objective exclusion criteria. Further research is required to investigate the potential for combining this approach with crowdsourcing or machine learning methodologies. Future directions should provide an intuitive platform for researchers to use one of the derived algorithms based on loss of sensitivity that is deemed acceptable and the desired work saving.

## Supplementary Information

**Additional file 1: Table S1.** Standards for Reporting of Diagnostic Accuracy Studies guidelines.

**Additional file 2: Table S2.** Examples of exclusion criteria.

**Additional file 3: Table S3.** Screening details of included systematic reviews.

**Additional file 4: Table S4.** Prevalence of exclusion criteria by set of systematic reviews.

**Additional file 5: Table S5.** Number of selected exclusion criteria by set of systematic reviews.

**Additional file 6: Table S6.** Performance of algorithms employing a single reviewer approach.

**Additional file 7: Table S7.** Steps to validate suggested algorithms in a local setting.

**Additional file 8: Figure S1.** Description of five algorithms developed based on the exploratory analysis. Otherwise refer to when the first reviewer retained the abstract or excluded it, but selected exclusion criteria did not satisfy the algorithm requirements.

**Additional file 9: Figure S2.** Loss of sensitivity using a single reviewer to a paper based on specific criteria (A) or for multiple reasons (B). Each circle is proportional in size the count of papers excluded based on this criterion. Error bars reflect 95%CI. In panel (A), the blue dotted line reflects the 1% threshold used in the algorithm development stage. Analysis is based on the systematic reviews in the derivation set (red) and the validation set (green).

## Data Availability

The datasets used and/or analysed during the current study are available from the corresponding author on reasonable request.

## Abbreviations

SD: Standard deviation
95%CI: 95% Confidence interval
SR: Systematic review
