# Supplementary material for: Successful incorporation of single reviewer assessments during systematic review screening: development and validation of sensitivity and work-saved of an algorithm that considers exclusion criteria and count
Source: Syst Rev. 2021 Apr 5;10:98. doi: 10.1186/s13643-021-01632-6 (PMC8020619; doi:10.1186/s13643-021-01632-6)
Supplement: Supplementary file 1 — Additional file 1: Table S1. Standards for Reporting of Diagnostic Accuracy Studies guidelines. [file 13643_2021_1632_MOESM1_ESM.docx]

**Additional table 1. Standards for Reporting of Diagnostic Accuracy Studies guidelines**

| **Section & Topic** | **No** | **Item** | **Reported on page #** |
| --- | --- | --- | --- |
| **TITLE OR ABSTRACT** | **1** | Identification as a study of diagnostic accuracy using at least one measure of accuracy (such as sensitivity, specificity, predictive values, or AUC) | 1 |
| **ABSTRACT** | **2** | Structured summary of study design, methods, results, and conclusions | 1 |
| **INTRODUCTION** |  |  |  |
|  | **3** | Scientific and clinical background, including the intended use and clinical role of the index test | 2 |
|  | **4** | Study objectives and hypotheses | 2 |
| **METHODS** |  |  |  |
| *Study design* | **5** | Whether data collection was planned before the index test and reference standard were performed (prospective study) or after (retrospective study) | 2 |
| *Participants* | **6** | Eligibility criteria | 2 |
|  | **7** | On what basis potentially eligible participants were identified (such as symptoms, results from previous tests, inclusion in registry) | 2 |
|  | **8** | Where and when potentially eligible participants were identified (setting, location and dates) | 2 |
|  | **9** | Whether participants formed a consecutive, random or convenience series | 2 |
| *Test methods* | **10a** | Index test, in sufficient detail to allow replication | 3 |
|  | **10b** | Reference standard, in sufficient detail to allow replication | 2 |
|  | **11** | Rationale for choosing the reference standard (if alternatives exist) | 2 |
|  | **12a** | Definition of and rationale for test positivity cut-offs or result categories of the index test, distinguishing pre-specified from exploratory | 3 |
|  | **12b** | Definition of and rationale for test positivity cut-offs or result categories of the reference standard, distinguishing pre-specified from exploratory | N/A |
|  | **13a** | Whether clinical information and reference standard results were available to the performers/readers of the index test | N/A |
|  | **13b** | Whether clinical information and index test results were available to the assessors of the reference standard | N/A |
| *Analysis* | **14** | Methods for estimating or comparing measures of diagnostic accuracy | 3 |
|  | **15** | How indeterminate index test or reference standard results were handled | 3 |
|  | **16** | How missing data on the index test and reference standard were handled | 2 |
|  | **17** | Any analyses of variability in diagnostic accuracy, distinguishing pre-specified from exploratory | 3 |
|  | **18** | Intended sample size and how it was determined | N/A |
| **RESULTS** |  |  |  |
| *Participants* | **19** | Flow of participants, using a diagram | Figure 1 |
|  | **20** | Baseline demographic and clinical characteristics of participants | Table 2 and Additional table 3 |
|  | **21a** | Distribution of severity of disease in those with the target condition | N/A |
|  | **21b** | Distribution of alternative diagnoses in those without the target condition | N/A |
|  | **22** | Time interval and any clinical interventions between index test and reference standard | N/A |
| *Test results* | **23** | Cross tabulation of the index test results (or their distribution) by the results of the reference standard | N/A |
|  | **24** | Estimates of diagnostic accuracy and their precision (such as 95% confidence intervals) | Figure 3 and Additional table 5 |
|  | **25** | Any adverse events from performing the index test or the reference standard | N/A |
| **DISCUSSION** |  |  |  |
|  | **26** | Study limitations, including sources of potential bias, statistical uncertainty, and generalisability | 8 |
|  | **27** | Implications for practice, including the intended use and clinical role of the index test | 8 |
| **OTHER INFORMATION** |  |  |  |
|  | **28** | Registration number and name of registry | N/A |
|  | **29** | Where the full study protocol can be accessed | N/A |
|  | **30** | Sources of funding and other support; role of funders | 9 |
