# Supplementary material for: Successful incorporation of single reviewer assessments during systematic review screening: development and validation of sensitivity and work-saved of an algorithm that considers exclusion criteria and count
Source: Syst Rev. 2021 Apr 5;10:98. doi: 10.1186/s13643-021-01632-6 (PMC8020619; doi:10.1186/s13643-021-01632-6)
Supplement: Supplementary file 2 — Additional file 2: Table S2. Examples of exclusion criteria. [file 13643_2021_1632_MOESM2_ESM.docx]

**Additional table 2. Examples of exclusion criteria.**

| Abstract (Conference) | - Study only published as a conference abstract |
| --- | --- |
| Age | - Study doesn’t target infant, neonates or children - Study includes only adults (>18 years of age) - Study includes both adults and children but pediatric data are not reported separately |
| Case report/series | - Study cohort is < 10 patients |
| Design | - Study is not a randomized controlled trial (RCT) - Study is not a retrospective or prospective cohort - Study design is not a randomized controlled trial, cohort comparison, or case series |
| Exposure | - Study doesn’t provide concussion education - Study doesn’t administer cyproheptadine as an appetite stimulant - Study doesn’t prescribe positive airway pressure therapy |
| Human | - Study targets cell line or animal research only |
| Language | - Study is not in English - Study is not in English, French, German, Russian, Polish, Arabic, or Hungarian - Study is published in a language other than English or French |
| Outcome | - Study doesn’t evaluate learning outcomes - Study doesn’t measure the increase in appetite - Study doesn’t assess adherence to positive airway pressure therapy |
| Population | - Study doesn’t include children or parents who received concussion education - Study population does not have sleep-disordered breathing - Study includes patients without native aortic root dilatation or aneurysm |
| Review | - Study is a systematic review - Study is a narrative review - Citation is an editorial, a commentary, or a response to letter |
| Setting | - Study doesn’t target patients in a clinical setting - Study only targets inpatient hospital setting - Trials conducted in a setting other than a pediatric intensive care unit |
