# Supplementary material for: Successful incorporation of single reviewer assessments during systematic review screening: development and validation of sensitivity and work-saved of an algorithm that considers exclusion criteria and count
Source: Syst Rev. 2021 Apr 5;10:98. doi: 10.1186/s13643-021-01632-6 (PMC8020619; doi:10.1186/s13643-021-01632-6)
Supplement: Supplementary file 3 — Additional file 3: Table S3. Screening details of included systematic reviews. [file 13643_2021_1632_MOESM3_ESM.docx]

**Additional table 3. Screening details of included systematic reviews.**

| **Systematic review** | **Type** | **Focus** | **Abstract screened^a^** | **Full-text screened** | **Eligible citations^b^** | **Reviewers** |
| --- | --- | --- | --- | --- | --- | --- |
| **Derivation Set** |  |  |  |  |  |  |
| Prematurity | Systematic review | Other | 257 | 28 | 10 | 2 |
| Radiology | Systematic review | Diagnosis | 503 | 89 | 28 | 4 |
| Nursing in emergency department | Systematic review | Therapeutic | 513 | 15 | 9 | 3 |
| Oncological pathology | Systematic review | Prognosis | 291 | 124 | 27 | 3 |
| Mental health | Scoping review | Other | 2114 | 173 | 54 | 5 |
| Pediatric respirology | Meta-analysis | Diagnosis | 277 | 115 | 27 | 2 |
| Sleep disorders | Meta-analysis | Diagnosis | 421 | 68 | 25 | 2 |
| Medical education | Systematic review | Other | 2469 | 441 | 68 | 4 |
| Nutrition | Systematic review | Therapeutic | 465 | 168 | 57 | 3 |
| Vaccination | Systematic review | Therapeutic | 2408 | 105 | 21 | 6 |
| **Subtotal** |  |  | **9718** | **1326** | **326** | **34** |
| **Validation Set** |  |  |  |  |  |  |
| Interventional cardiology | Systematic review | Therapeutic | 690 | 216 | 6 | 6 |
| Ethics in general pediatrics | Scoping review | Other | 862 | 362 | 102 | 2 |
| Gastroenterology and general surgery | Meta-analysis | Therapeutic | 585 | 347 | 159 | 5 |
| Critical care | Scoping review | Other | 286 | 17 | 6 | 2 |
| Obstetrics pathology | Systematic review | Other | 525 | 233 | 73 | 7 |
| Personal protective equipment | Living meta-analysis | Therapeutic | 417 | 16 | 10 | 3 |
| Perinatal infections | Systematic review | Prognosis | 9648 | 453 | 69 | 9 |
| Pediatric general surgery | Meta-analysis | Therapeutic | 232 | 225 | 113 | 4 |
| Gastroenterological pathology | Systematic review | Diagnosis | 888 | 134 | 35 | 5 |
| Heat decontamination | Living meta-analysis | Therapeutic | 416 | 19 | 16 | 7 |
| Emergency medical education | Systematic review | Therapeutic | 1549 | 74 | 19 | 3 |
| Respirology and radiology | Systematic review | Diagnosis | 801 | 166 | 23 | 4 |
| Surgical masks | Living systematic review | Therapeutic | 1874 | 33 | 2 | 12 |
| Ultraviolet decontamination | Living meta-analysis | Therapeutic | 1108 | 55 | 15 | 13 |
| **Subtotal** |  |  | **19881** | **2350** | **648** | **82** |
| **Total** |  |  | **29599** | **3676** | **974** | **116** |

^a^ Total number of citations identified by the search strategy.

^b^ Eligible citations as identified by the investigative team (ie, true positives).
