# Supplementary material for: Successful incorporation of single reviewer assessments during systematic review screening: development and validation of sensitivity and work-saved of an algorithm that considers exclusion criteria and count
Source: Syst Rev. 2021 Apr 5;10:98. doi: 10.1186/s13643-021-01632-6 (PMC8020619; doi:10.1186/s13643-021-01632-6)
Supplement: Supplementary file 4 — Additional file 4: Table S4. Prevalence of exclusion criteria by set of systematic reviews. [file 13643_2021_1632_MOESM4_ESM.docx]

**Additional table 4. Prevalence of exclusion criteria by set of systematic reviews.**

| Exclusion | Count of SRs | Count of Papers^a^ | Concordance | Loss of Sensitivity  Mean [95%CI] |
| --- | --- | --- | --- | --- |
| **Derivation** |  |  |  |  |
| Abstract (Conference) | 5 | 791 | 90.6% | 0.0% [0.0% - 1.2%] |
| Age | 5 | 142 | 83.8% | 0.0% [0.0% - 1.2%] |
| Case report/series | 6 | 142 | 92.3% | 0.0% [0.0% - 1.2%] |
| Design | 6 | 357 | 89.4% | 0.3% [0.1% - 1.7%] |
| Exposure | 9 | 4632 | 84.6% | 3.2% [1.8% - 5.7%] |
| Human | 6 | 735 | 99.9% | 0.0% [0.0% - 1.2%] |
| Language | 2 | 119 | 86.6% | 0.2% [0.0% - 1.5%] |
| Outcome | 10 | 2792 | 87.1% | 1.8% [0.8% - 4.0%] |
| Population | 9 | 4700 | 89.4% | 4.1% [2.5% - 6.9%] |
| Review | 7 | 362 | 83.1% | 0.0% [0.0% - 1.2%] |
| Setting | 7 | 766 | 84.1% | 0.3% [0.1% - 1.7%] |
|  |  |  |  |  |
| **Validation** |  |  |  |  |
| Abstract (Conference) | 1 | 1078 | 99.9% | 0.1% [0.0% - 0.7%] |
| Age | 6 | 518 | 89.0% | 0.2% [0.0% - 0.9%] |
| Design | 8 | 3740 | 95.2% | 0.6% [0.2% - 1.6%] |
| Exposure | 8 | 12449 | 97.7% | 1.2% [0.6% - 2.4%] |
| Language | 7 | 170 | 91.2% | 0.0% [0.0% - 0.6%] |
| Outcome | 11 | 4744 | 96.2% | 0.6% [0.2% - 1.6%] |
| Population | 7 | 6171 | 97.5% | 0.8% [0.3% - 1.8%] |
| Review | 6 | 1243 | 89.6% | 0.4% [0.1% - 1.2%] |
| Setting | 3 | 1054 | 95.0% | 0.1% [0.0% - 0.7%] |

^a^ Number of papers where the criterion was selected.
