# Supplementary material for: Successful incorporation of single reviewer assessments during systematic review screening: development and validation of sensitivity and work-saved of an algorithm that considers exclusion criteria and count
Source: Syst Rev. 2021 Apr 5;10:98. doi: 10.1186/s13643-021-01632-6 (PMC8020619; doi:10.1186/s13643-021-01632-6)
Supplement: Supplementary file 5 — Additional file 5: Table S5. Number of selected exclusion criteria by set of systematic reviews. [file 13643_2021_1632_MOESM5_ESM.docx]

**Additional table 5. Number of selected exclusion criteria by set of systematic reviews.**

| Exclusion | Count of SRs | Count of Papers^a^ | Concordance | Loss of Sensitivity  Mean [95%CI] |
| --- | --- | --- | --- | --- |
| **Derivation** |  |  |  |  |
| ≥ 1 | 10 | 8021 | 82.0% | 8.4% [5.9% - 12.0%] |
| ≥ 2 | 8 | 3674 | 89.6% | 1.2% [0.5% - 3.1%] |
| ≥ 3 | 8 | 1449 | 89.7% | 0.5% [0.1% - 2.0%] |
| ≥ 4 | 6 | 296 | 95.6% | 0.0% [0.0% - 1.2%] |
|  |  |  |  |  |
| **Validation** |  |  |  |  |
| ≥ 1 | 14 | 17507 | 94.6% | 3.6% [2.4% - 5.4%] |
| ≥ 2 | 14 | 9481 | 98.3% | 0.3% [0.1% - 1.1%] |
| ≥ 3 | 10 | 2027 | 98.2% | 0.2% [0.0% - 0.9%] |
| ≥ 4 | 7 | 145 | 97.2% | 0.0% [0.0% - 0.6%] |

^a^ Number of papers where the criterion was selected.
