# Supplementary material for: Successful incorporation of single reviewer assessments during systematic review screening: development and validation of sensitivity and work-saved of an algorithm that considers exclusion criteria and count
Source: Syst Rev. 2021 Apr 5;10:98. doi: 10.1186/s13643-021-01632-6 (PMC8020619; doi:10.1186/s13643-021-01632-6)
Supplement: Supplementary file 6 — Additional file 6: Table S6. Performance of algorithms employing a single reviewer approach. [file 13643_2021_1632_MOESM6_ESM.docx]

**Additional table 6. Performance of algorithms employing a single reviewer approach.**

| Algorithm | Loss of Sensitivity (%)^a^  Mean [95%CI] | | Work-saved (%)^b^  Mean [95%CI] | |
| --- | --- | --- | --- | --- |
|  | Derivation | Validation | Derivation | Validation |
| 1 | 1.2% [0.5% - 3.1%] | 0.3% [0.1% - 1.2%] | 25.2% [24.3% - 26.1%] | 32.0% [31.4% - 32.7%] |
| 2 | 0.8% [0.2% - 2.4%] | 1.2% [0.6% - 2.5%] | 22.2% [21.3% - 23.0%] | 27.5% [26.8% - 28.1%] |
| 3 | 0.0% [0.0% - 1.2%] | 0.6% [0.3% - 1.7%] | 14.8% [14.1% - 15.6%] | 10.5% [10.1% - 10.9%] |
| 4 | 1.9% [0.8% - 4.0%] | 1.3% [0.7% - 2.6%] | 37.7% [36.7% - 38.7%] | 48.2% [47.5% - 48.9%] |
| 5 | 1.2% [0.5% - 3.1%] | 0.8% [0.4% - 1.9%] | 33.6% [32.6% - 34.5%] | 39.8% [39.1% - 40.5%] |

^a^ Loss of sensitivity is the percentage of eligible citations incorrectly excluded by the algorithm at the abstract level among all eligible citations.

^b^ Work-saved is defined as the percentage of all citations that were excluded by the algorithm without requiring a second assessment by the investigative team.
