# Supplementary material for: Successful incorporation of single reviewer assessments during systematic review screening: development and validation of sensitivity and work-saved of an algorithm that considers exclusion criteria and count
Source: Syst Rev. 2021 Apr 5;10:98. doi: 10.1186/s13643-021-01632-6 (PMC8020619; doi:10.1186/s13643-021-01632-6)
Supplement: Supplementary file 7 — Additional file 7: Table S7. Steps to validate suggested algorithms in a local setting. [file 13643_2021_1632_MOESM7_ESM.docx]

**Additional table 7. Steps to validate suggested algorithms in a local setting.**

| 1. Stratify the exclusion criteria in the categories as described in this study. |
| --- |
| 1. Screen a proportion of the citations (e.g., 10%) using the gold-standard dual-reviewer approach. Ensure that reviewers are selecting all applicable exclusion criteria when excluding a citation. |
| 1. Evaluate the proportion of eligible citations that would have been missed when the first reviewer selected multiple reasons of exclusion or selected one of the specific criteria highlighted in the five algorithms (see Additional figure 1). |
| 1. Determine the potential work-saved for the second reviewer by employing one of the five algorithms. |
| 1. Determine which algorithm maintains an acceptable sensitivity (e.g., above 95%, or equals 100%), while achieving the highest potential work-saved. |
| 1. Screen the remaining citations (i.e., the other 90%) while applying the selected algorithm. Citations excluded without satisfying the algorithm’s conditions will need to be screened by two reviewers. |
| 1. To validate this approach locally and more broadly, researchers can apply these algorithms (retrospectively or prospectively) to some systematic reviews and compare to the gold-standard. Using 3-5 systematic reviews covering a variety of topics and methodologies could be sufficient to highlight whether these algorithms are valid in their local setting. |
