# Supplementary material for: Successful incorporation of single reviewer assessments during systematic review screening: development and validation of sensitivity and work-saved of an algorithm that considers exclusion criteria and count
Source: Syst Rev. 2021 Apr 5;10:98. doi: 10.1186/s13643-021-01632-6 (PMC8020619; doi:10.1186/s13643-021-01632-6)
Supplement: Supplementary file 8 — Additional file 8: Figure S1. Description of five algorithms developed based on the exploratory analysis. Otherwise refer to when the first reviewer retained the abstract or excluded it, but selected exclusion criteria did not satisfy the algorithm requirements. [file 13643_2021_1632_MOESM8_ESM.docx]

**Additional figure 1. Description of five algorithms developed based on the exploratory analysis.** Otherwise refer to when the first reviewer retained the abstract or excluded it, but selected exclusion criteria did not satisfy the algorithm requirements.
