# Supplementary material for: Successful incorporation of single reviewer assessments during systematic review screening: development and validation of sensitivity and work-saved of an algorithm that considers exclusion criteria and count
Source: Syst Rev. 2021 Apr 5;10:98. doi: 10.1186/s13643-021-01632-6 (PMC8020619; doi:10.1186/s13643-021-01632-6)
Supplement: Supplementary file 9 — Additional file 9: Figure S2. Loss of sensitivity using a single reviewer to a paper based on specific criteria (A) or for multiple reasons (B). Each circle is proportional in size the count of papers excluded based on this criterion. Error bars reflect 95%CI. In panel (A), the blue dotted line reflects the 1% threshold used in the algorithm development stage. Analysis is based on the systematic reviews in the derivation set (red) and the validation set (green). [file 13643_2021_1632_MOESM9_ESM.docx]

**Additional figure 2. Loss of sensitivity using a single reviewer to a paper based on specific criteria (A) or for multiple reasons (B).** Error bars reflect 95%CI. In panel (A), the blue dotted line reflects the 1% threshold used in the algorithm development stage. Analysis is based on the systematic reviews in the derivation set (red circle) and the validation set (green square).
